# Serine 307 on insulin receptor substrate 1 is required for SOCS3 and TNF-α signaling in the rMC-1 cell line

**Published:** 2014-10-23

**Authors:** Youde Jiang, Subrata K. Biswas, Jena J. Steinle

**Affiliations:** 1 Department of Ophthalmology, University of Tennessee Health Science Center, Memphis, TN; 2 Department of Anatomy/Neurobiology, University of Tennessee Health Science Center, Memphis, TN; 3 Department of Pharmaceutical Sciences, University of Tennessee Health Science Center, Memphis, TN

## Abstract

**Purpose:**

To establish the key insulin receptor substrate 1 (IRS-1) structural elements required in this insulin regulatory pathway, we investigated the effects of substituting alanine for serine 307 in IRS-1 on the ability of tumor necrosis factor-α (TNF-α) and a related mediator, suppressor of cytokine signaling 3 (SOCS3), to phosphorylate IRS-1 and regulate insulin signaling in the rat retinal Müller cell (rMC-1) cell line.

**Methods:**

rMC-1 cells were grown in normal (5 mM) or high (25 mM) glucose medium and transfected with either normal IRS-1^Ser307^plasmid or a mutated IRS-1^Ser307Ala^ plasmid. Cells were also treated with recombinant TNF-α or SOCS3 to induce increased levels of these proteins.

**Results:**

In cells with IRS-1^Ser307Ala^, TNF-α and SOCS3 failed to phosphorylate IRS-1. Likewise, resulting downstream effects, including changes in phosphorylation of insulin receptor^Tyr960^, antiapoptotic Akt phosphorylation, and proapoptotic cleavage of caspase 3 were also blocked. We also report for the first time that SOCS3 and TNF-α are reciprocally stimulatory leading to a mutual enhancement of levels of both factors, thus forming a potential positive feedback loop that contributes to insulin receptor resistance.

**Conclusions:**

Increases in TNF-α and SOCS3 are triggered by high glucose and through reciprocal stimulation of expression of these two factors, which in turn could be major drivers of insulin resistance and related cell death. The demonstration that a single phosphorylation site is key for these pathways suggests that drugs targeted to this site might be effective in protecting against diabetic damage to the retina.

## Introduction

Diabetes produces several physiologic and metabolic changes in the retina, many of which are still poorly understood. One of the first cell types to be altered in response to high glucose is the Müller cell [1]. The expression of tumor necrosis factor-α (TNF-α) [2], along with the stress marker, glial fibrillary acidic protein [3], increases in Müller cells early in response to excess glucose. In previous work, we have shown that TNF-α is highly involved in regulating insulin signaling in retinal Müller cells [4], such that increased TNF-α inhibits normal insulin signal transduction in these cells. One of the pathways by which TNF-α can inhibit insulin signaling is through phosphorylation of insulin receptor substrate 1 (IRS-1) on serine 307 [5,6].

In addition to regulating IRS-1, TNF-α can also regulate insulin signal transduction through increasing levels of suppressor of cytokine signaling 3 (SOCS3) [7]. SOCS3 is reported to inhibit insulin signaling by multiple potential mechanisms, including increased phosphorylation of insulin receptor on tyrosine 960 (IR^Tyr960^), which inhibits the interaction between insulin receptor and IRS-1 [8]. In addition, SOCS3 also can lead to ubiquitinization of IRS-1 to block normal insulin signaling [9]. Additionally, some have reported that SOCS3 inhibition of Stat5B can also inhibit insulin’s ability to activate IRS-1 in Cos7 cells [10]. It is unclear whether SOCS3 can regulate insulin signal transduction through the phosphorylation of IRS-1 on serine 307, similar to TNF-α [6]. Additionally, it is also unknown whether SOCS3 can stimulate increased TNF-α levels. The exact interaction between TNF-α and SOCS3 in regulating insulin receptor signal transduction may offer new clues for diabetic retinopathy therapeutics.

Since TNF-α and SOCS3 can negatively regulate insulin receptor signaling through IRS-1 in retinal endothelial cells [11], we wanted to determine whether mutation of the serine 307 site on IRS-1 could block the inhibitory actions of TNF-α and SOCS3 on insulin signaling, and thus prevent apoptosis of rat retinal Müller cells (rMC-1) cells. Because we have previously published work in these cells and insulin signaling [4,12], we compared rMC-1 cells grown in normal glucose and high glucose after transfection with plasmid of normal IRS-1 or a mutant form of IRS-1 where serine 307 is mutated to an alanine for this study. To further examine the direct effects of TNF-α and SOCS3 on IRS-1 signaling, we also treated with recombinant TNF-α or SOCS3 to create an excess of these factors following transfection of cells with IRS-1 plasmid or mutant plasmid.

## Methods

### Rat retinal Müller cell culture

Rat retinal Müller cells (courtesy of Vijay Sarthy, Northwestern University) were grown in 5 mM or 25 mM glucose Dulbecco's Modified Eagle Medium (DMEM; HyClone Laboratories, Logan, UT). We chose to use this model as we have previously published the effects of β-adrenergic receptor agonists on insulin signaling in these cells [4]. Medium was supplemented with 10% fetal bovine serum (FBS) and antibiotics. Cells were cultured to 80% confluency (2–4 days), and then the cells were starved for 18–24 h by reduction to 2% FBS in the growth medium to eliminate any residual growth factors in the serum. We chose to reduce serum to 2% rather than complete starvation to eliminate activation of apoptotic pathways. Additionally, we have used this method in the past for measurements of TNF-α and insulin pathways [4].

### Cell treatments

Some cells were treated with TNF-α (5 ng/ml, R&D Systems) for 30 min, while additional cells were treated with recombinant SOCS3 (500 ng/ml, Abnova, through Fisher Scientific, Pittsburgh, PA) for 60 min. For the treatments with plasmids, plasmid transfection preceded treatment with SOCS3 or TNF-α. For overexpression of normal IRS-1 and its mutant IRS-1^Ser307Ala^ genes in rMC-1 cells, the cells were passaged and cultured to reach 80% confluency. Cells were then transiently transfected with plasmids expressing either wild-type IRS-1 or mutant IRS-1 (Ser307Ala) genes using GeneMute transfection reagent (SignaGen Laboratories, Rockville, MD) according to the manufacturer’s instructions.

### Plasmid

Rat IRS-1 plasmid obtained from Addgene (Addgene plasmid 11,027, in bacterial expression vector, Cambridge, MA) was used to generate the rIRS-1^Ser307Ala^ mutant plasmid as described below. The native and mutant rIRS-1 genes were then sub-cloned in mammalian expression vector pCMV-M1 (Addgene plasmid 23,007, Cambridge, MA) by double digestion with SacI and HindIII (Thermo Scientific, Pittsburgh, PA), gel purification of the digestion fragments followed by ligation with T4 DNA ligase (Promega, through Fisher Scientific, Pittsburgh, PA).

### Site-directed mutagenesis for generation of rIRS-1^Ser307Ala^ plasmids

Mutagenesis was performed using the QuickChange II XL site-directed mutagenesis kit (Stratagene, Santa Clara, CA). Briefly, a pair of primers with the desired mutations was designed using Stratagene’s primer design software in such a way that they introduce an alanine instead of serine at the 307 position of the IRS-1 gene while using the plasmid as a template in thermal cycling. *PfuUltra* high-fidelity DNA polymerase (Stratagene) was used for PCR cycling under the following conditions 95 °C for 1 min followed by 18 cycles each consisting of 95 °C for 50 s, 60 °C for 50 s and 68 °C for 1 min per kb of plasmid length. Finally, 7 min at 68 °C was allowed for final extension. The PCR product was then digested with Dpn-I to remove the methylated parental DNA. The nicked vector DNA incorporating the desired mutations was then transformed into XL10-Gold ultracompetent cells followed by plating on selective Luria broth (LB) plates. Mutation was verified with DNA sequencing, and protein levels were confirmed with enzyme-linked immunosorbent assay (ELISA).

### Cloning method for generation of mammalian expression vectors

Rat IRS-1 plasmid and the respective mutant plasmid were generated by sub-cloning the genes from cloning vectors into mammalian expression vectors following the instructions of Invitrogen’s pcDNA Gateway Directional TOPO expression kit (Invitrogen, Carlsbad, CA). Briefly, forward PCR primers were designed with CACC at the 5′ end of the primer followed by a Kozak translation initiation sequence ATG. Reverse primers were designed conventionally with special care not to include a sequence complementary to the overhang sequence GTGG at the 5′ end. A blunt-end PCR product was produced using a thermostable proofreading DNA polymerase (*PfuUltra* high-fidelity from Stratagene). The PCR product was gel purified and cloned in TOPO vector pcDNA 3.2. The cloning reaction was then used to transform One Shot TOP10 chemically competent *E. coli*, and the bacterial culture was spread on the prewarmed selective plate. Expression plasmids were isolated from single colonies, and the plasmids were analyzed to confirm the presence and correct orientation of the insert with restriction enzyme digestion and DNA sequencing.

### Western blot analysis

After appropriate treatments and rinsing with cold PBS PBS (1X; 137 mM NaCl, 2.7 mM KCl, 8 mM Na_2_HPO_4_, and 2 mM KH_2_PO_4_; pH 7.4), rMC-1 cells were treated with lysis buffer containing protease and phosphatase inhibitors and scraped into the tubes. Equal amounts of protein from cell lysates were separated on the precast tris-glycine gel (Invitrogen), and blotted onto a nitrocellulose membrane. After blocking in TBST (10 mM Tris-HCl buffer, pH 8.0, 150 mM NaCl, 0.1% Tween-20) and 5% (w/v) bovine albumin serum (BSA), the membrane was treated with appropriate primary antibodies followed by incubation with secondary antibodies labeled with horseradish peroxidase. Antigen-antibody complexes were detected with the chemiluminescence reagent kit (Thermo Scientific, Pittsburgh, PA). Primary antibodies used were phosphorylated Akt (Serine 473), Akt, total IRS-1, SOCS3 (all purchased from Cell Signaling, Danvers, MA), beta actin (Santa Cruz), and insulin receptor (Tyr 960, Cell Applications). Western blot analyses of proteins of interest were compared to beta actin levels, and a ratio is presented.

### ELISA analysis

A cleaved caspase 3 ELISA (Cell Signaling, Danvers, MA) was used to measure levels of the active apoptotic marker in rMC-1 cells, while phosphorylation of IRS-1 on serine 307 was measured with ELISA (Cell Signaling, Danvers, MA) with equal protein loaded (50 μg) for both ELISAs to allow for analyses using optical density measurements. By loading equal protein into the cleaved caspase 3 and IRS-1^Ser307^ ELISA, we eliminated bias for transfection efficiency or cell numbers. TNF-α protein concentrations in the cell lysates were measured using a TNF-α ELISA (ThermoFisher, Pittsburgh, PA).

### Statistical analysis

Statistical significance was determined using a one-way ANOVA with Student Newman Keul’s post-hoc test with p<0.05 taken as statistically significant. Data are shown as means± standard error of the mean (SEM). For western blots, a representative blot is provided.

## Results

### rMC-1 cells expressed GFAP and CRALBP

**Figure 1:**
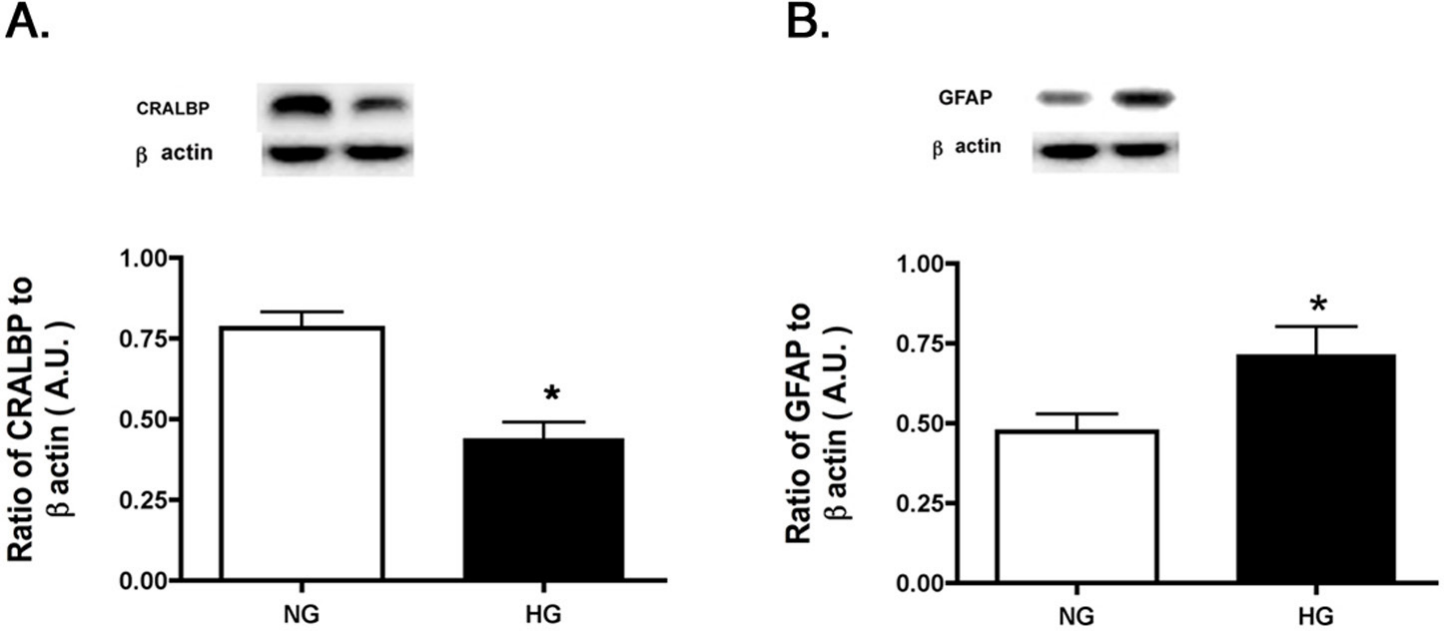
Western blot data on rMC-1 cells grown in normal glucose (NG) or high glucose (HG) to measure CRALBP and GFAP using retinal cell lysates. Data demonstrate that the cells used for these experiments still express key Müller cell proteins. *p<0.05 versus normal glucose.

To confirm that the *rMC-1* cells used for these studies express key markers of retinal Müller cells, we performed western blotting for two key Müller cell marker proteins, glial fibrillary acidic protein (GFAP) and cellular retinaldehyde-binding protein (CRALBP), as reported in the original description of this cell line in 1998 [13] (Figure 1). GFAP and CRALBP protein levels were measured in rMC-1 cells starved in 2% serum overnight to reflect the standard conditions used for the remainder of experiments presented below. Figure 1 shows that CRALBP and GFAP are expressed in this passage of rMC-1 cells and demonstrated that high glucose increased GFAP levels [14]. Based on these results and the extensive characterization of the rMC-1 in previous studies by our laboratory and others [1,13,14], we chose to use these cells as a valid model system for assessing IRS-1/TNF-α signaling in Müller cells.

### Lack of TNF-α stimulated phosphorylation of mutated IRS-1^Ser307Ala^

**Figure 2:**
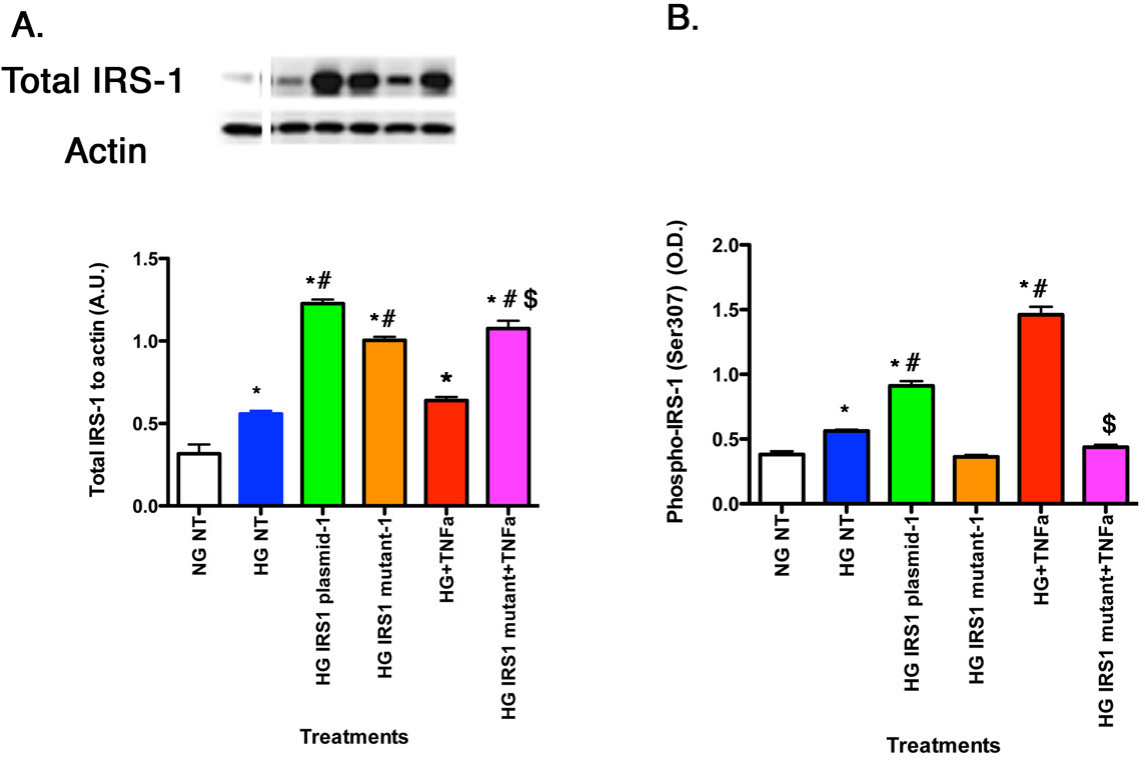
For all panels, retinal Müller cells were grown in normal glucose (NG) or high glucose (HG) and transfected with an IRS-1 plasmid or a mutated IRS-1 plasmid and treated with TNF-α or TNF-α+mutant (Panels **A**–**B**) with retinal cell lysates used for all experiments. **A**: The control demonstrates successful transfection of the normal IRS-1 plasmid and mutant IRS-1 plasmid. **B**: The ELISA results for decreased phosphorylation of IRS-1 on serine 307 when TNF-α is added with the mutant. *p<0.05 versus NG non-treated; #p<0.05 versus HG non-treated; $p<0.05 versus HG TNF-α. n=5 for all groups. Data are mean±standard error of the mean (SEM).

To test our hypothesis that IRS-1^Ser307^ is required for TNF-α actions on rMC-1 cells, we generated a mutant IRS-1 in which alanine was substituted for serine 307. Cells were then transfected with either normal IRS-1^Ser307^ plasmid or mutant IRS-1^Ser307Ala^ plasmid. We confirmed that treatment with exogenous TNF-α in normal (NG) and high glucose (HG) led to a significant increase in TNF-α levels with data that demonstrate exogenous TNF-α treatment increased TNF-α levels in rMC-1 cells by approximately 3.5 fold, measured with ELISA. Western blot confirmed that transfection of the normal plasmid and the mutant plasmid resulted in increased levels of total IRS-1 in all groups undergoing transfection or treated with high glucose (Figure 2A). Additionally, we tested whether TNF-α could induce phosphorylation of IRS-1 if serine 307 was mutated. We found that cells transfected with normal IRS-1 plasmid had increased phosphorylation of IRS-1^Ser307^ compared to the high glucose only treatment; whereas cells transfected with mutated IRS-1 plasmid had no such increase in phosphorylation, even with exogenous TNF-α added. This result was expected since the mutant form of IRS-1^Ser307Ala^ lacks the potential phosphorylation site hypothesized to be the target of TNF-α-mediated actions. Additionally, the data helped rule out the possibility that other potential phosphorylation sites could be activated by TNF-α as a result of mutant transfection. Based on these findings, we conclude that IRS-1^Ser307^ is key for TNF-α actions in rMC-1 cells.

### TNF-α regulated Akt through IRS-1^Ser307^, leading to increased cleaved caspase 3

**Figure 3:**
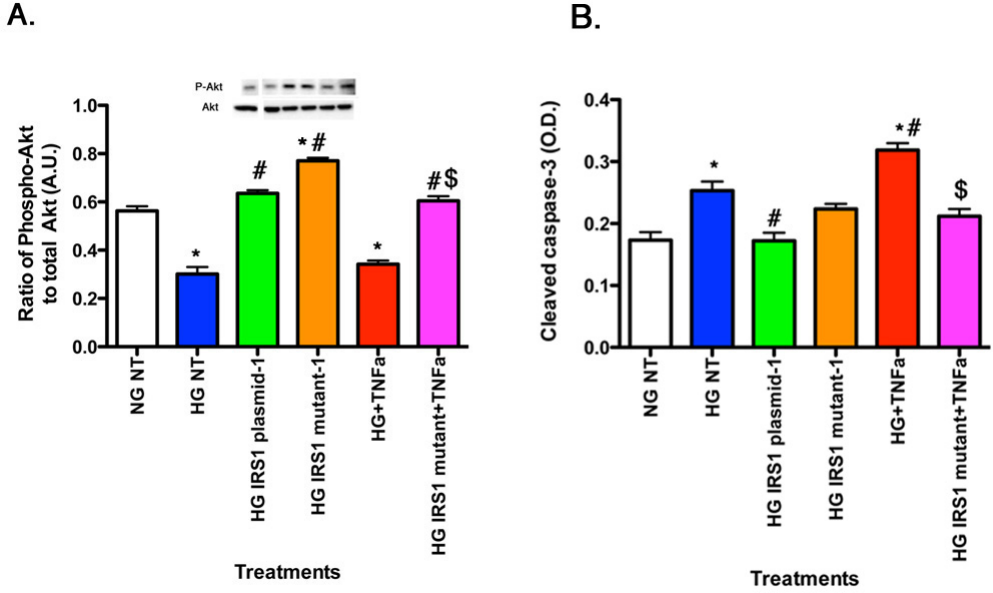
For all panels, rMC-1 cells were grown in normal glucose (NG) or high glucose (HG) and transfected with an IRS-1 plasmid or a mutated IRS-1 plasmid and treated with TNF-α or TNF-α+mutant with retinal cell lysates used for all experiments. Data show that TNF-α regulates Akt (**A**) through IRS-1^Ser307^, leading to decreased cleaved caspase 3 (**B**) when IRS-1^Ser307^ is mutated. *p<0.05 versus NG non-treated; #p<0.05 versus HG non-treated; $p<0.05 versus HG TNF-α. n=5 for all groups. Data are mean±standard error of the mean (SEM).

rMC-1 cells grown in high glucose had decreased Akt phosphorylation (Figure 3A) and increased cleaved caspase 3 levels (Figure 3B), indicating stimulated apoptosis compared to cells grown in normal glucose. TNF-α alone decreased Akt phosphorylation (Figure 3A) and increased cleavage of caspase 3 (Figure 3B). In cells transfected with mutant IRS-1*^Ser307Ala^* and treated with TNF-α, Akt levels remained at levels similar to normal glucose cells and cleaved caspase 3 remained low, demonstrating that TNF-α regulated Akt and apoptosis through IRS-1^Ser307^.

### TNF-α regulated SOCS3 and IR^Tyr960^ through IRS-1^Ser307^ pathways

**Figure 4:**
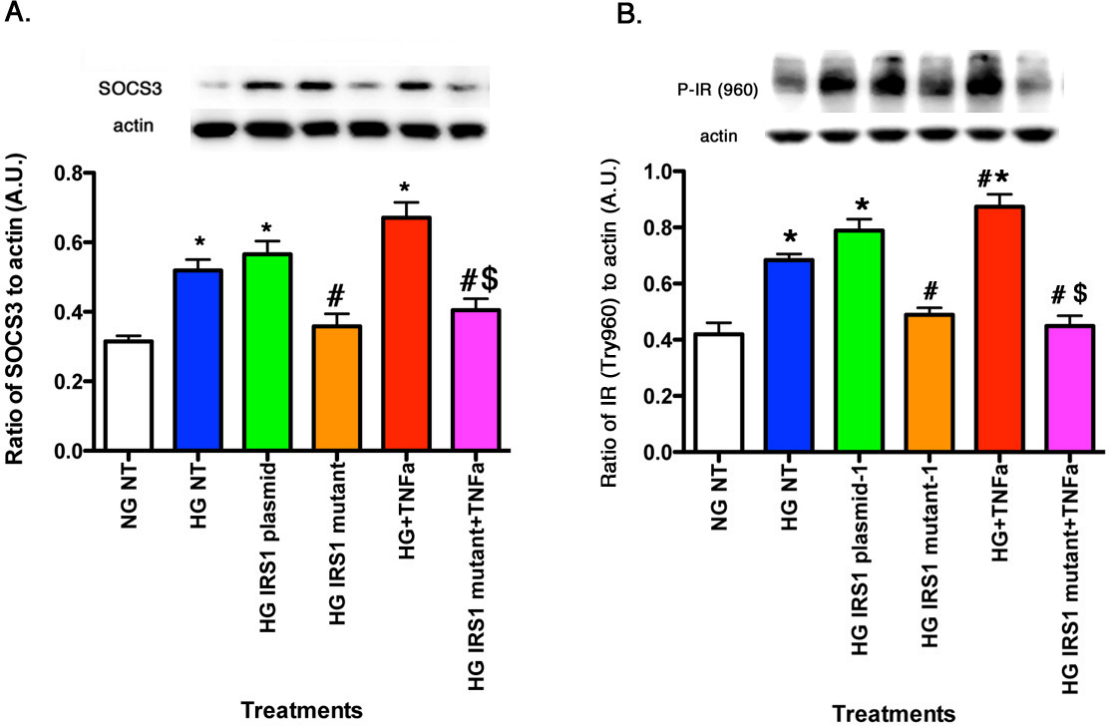
For all panels, rMC-1 cells were grown in normal glucose (NG) or high glucose (HG) and transfected with an IRS-1 plasmid or a mutated IRS-1 plasmid and treated with TNF-α or TNF-α+mutant with retinal cell lysates used for all experiments. **A**: The actions of TNF-α on levels of SOCS3. **B**: TNF-α requires IRS-1^Ser307^ to regulate IR^Tyr960^. *p<0.05 versus NG non-treated; #p<0.05 versus HG non-treated; $p<0.05 versus HG TNF-α. n=5 for all groups. Data are mean±standard error of the mean (SEM).

In addition to effects on the IRS-1 and apoptotic pathways, TNF-α is also known to activate SOCS3 in response to stressors, such as high glucose [7]. In turn, SOCS3 may likewise inhibit insulin signaling through several pathways including phosphorylation of the insulin receptor, specifically IR^Tyr960^. Figure 4A showed that TNF-α alone increased SOCS3 levels in rMC-1 cells. This response was eliminated when TNF-α was added with IRS-1^Ser307Ala^ mutants, suggesting that TNF-α actions on SOCS3 may require this IRS-1 site. Since SOCS3 preferentially phosphorylates the insulin receptor on tyrosine 960, we also wanted to investigate TNF-α actions on this site. High glucose alone increased phosphorylation of IR^Tyr960^, which was further enhanced with TNF-α treatment (Figure 4B). However, when TNF-α was added with mutant IRS-1, phosphorylation of IR^Tyr960^ levels were significant reduced compared to high glucose only cells, suggesting that serine 307 on IRS-1 is key to TNF-α actions in rMC-1 cells.

### SOCS3 regulated TNF-α levels as well as phosphorylation of IRS-1^Ser307^

**Figure 5:**
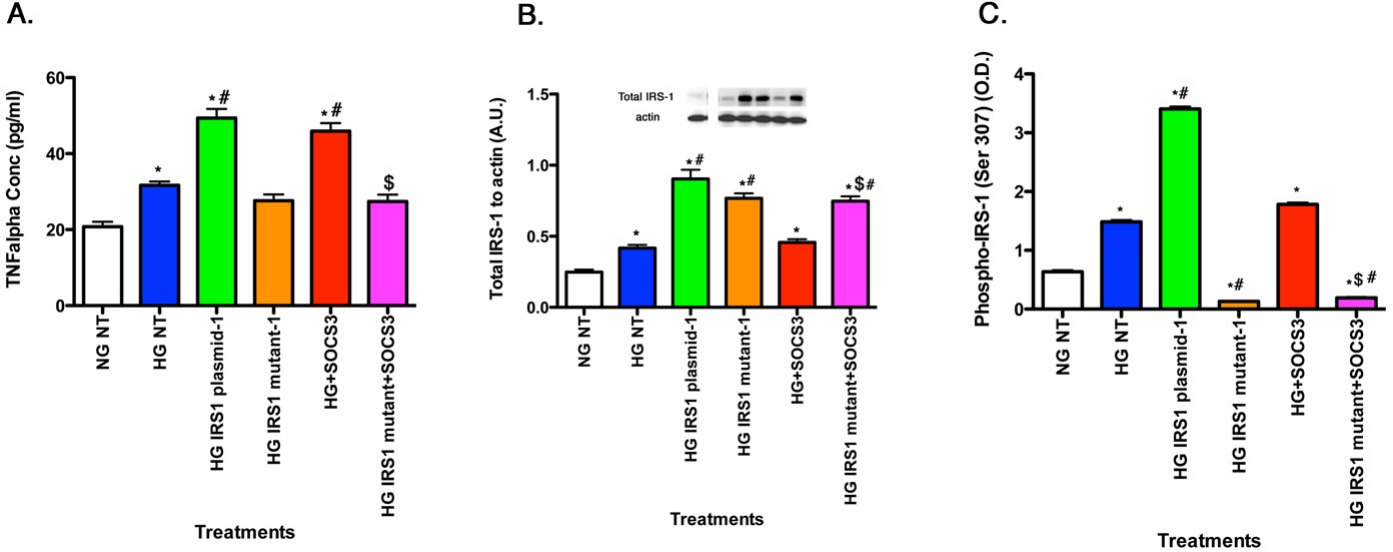
For all panels, rMC-1 cells were grown in normal glucose (NG) or high glucose (HG) and transfected with an IRS-1 plasmid or a mutated IRS-1 plasmid and treated with SOCS3 and SOCS3 with mutant plasmid with retinal cell lysates used for all experiments. **A**: SOCS3 regulates TNF-α levels in rMC-1 cells. **B**: The control demonstrates successful transfection of the plasmid and the mutant. **C**: ELISA data show that SOCS3 decreased phosphorylation of IRS-1 on serine 307 when added with the mutant. *p<0.05 versus NG non-treated; #p<0.05 versus HG non-treated; $p<0.05 versus HG SOCS3. n=5 for all groups. Data are mean±standard error of the mean (SEM).

There is considerable evidence in the literature that TNF-α regulates SOCS3 [7]; however, much less is known regarding the possible SOCS3 regulation of TNF-α. Figure 5A shows that exogenous SOCS3 significantly increased TNF-α levels (Figure 5A, red bar), which occurs through activation of the IRS-1^Ser307^ site, since cells transfected with mutant plasmid and exogenous SOCS3 did not have increased TNF-α levels when compared to SOCS3 alone (Figure 5A, magenta bar). SOCS3 significantly increased levels of total IRS-1 (Figure 5B, red bar) and IRS-1^Ser307^ (Figure 5C, red bar); this response was completely eliminated in the cells transfected with the IRS-1^Ser307Ala^ mutant before treatment with exogenous SOCS3 (Figure 5C, magenta bar). The normal IRS-1 plasmid still increased IRS-1^Ser307^ phosphorylation, but the mutation was effective in blocking all SOCS3 responses (Figure 5C).

### Lack of TNF-α- and SOCS3-dependent phosphorylation of IR^Tyr960^ in cells with mutated IRS-1^Ser307Ala^

**Figure 6:**
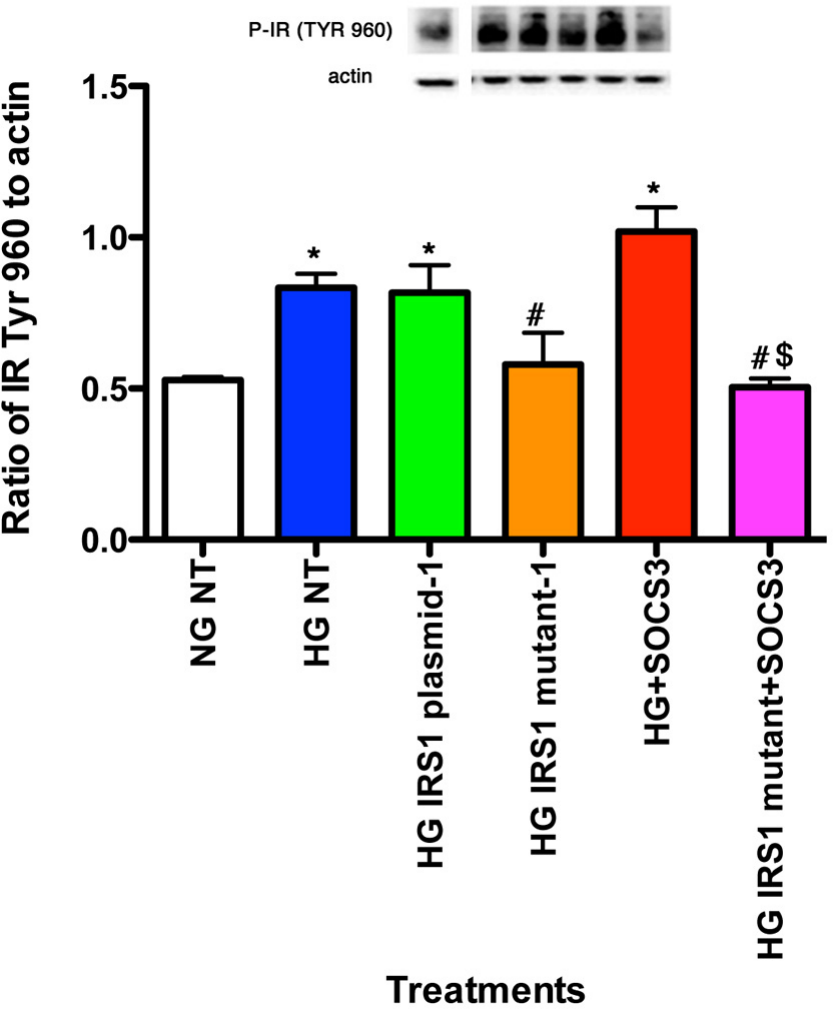
Data show rMC-1 cells were grown in normal glucose (NG) or high glucose (HG) and transfected with an IRS-1 plasmid or a mutated IRS-1 plasmid and treated with SOCS3 (SOCS3) or SOCS3+mutant. Data shows that SOCS3 requires IRS-1^Ser307^ to regulate IR^Tyr960^. *p<0.05 versus NG non-treated; #p<0.05 versus HG non-treated; $p<0.05 versus HG SOCS3. n=5 for all groups. Data are mean±standard error of the mean (SEM).

Adding exogenous SOCS3 to rMC-1 cells increased SOCS3 levels in untreated cells cultured in high glucose by approximately 30%, while increasing SOCS3 levels in all other high glucose treatment groups by greater than twofold. Figure 6 shows that SOCS3 required active IRS-1^Ser307^ to regulate IR^Tyr960^. SOCS3 alone significantly increased IR^Tyr960^ (Figure 6, red bar), which is in agreement with the literature [15]. The result of this increase was a disruption in insulin receptor actions. However, when rMC-1 cells were transfected with the mutant IRS1^Ser307Ala^, SOCS3 was unable to increase IR^Tyr960^ (Figure 6, magenta bar), suggesting that serine 307 is key to SOCS3 actions on IR^Tyr960^.

### SOCS3 regulated Akt and cleavage of caspase 3 through IRS-1^Ser307^

**Figure 7:**
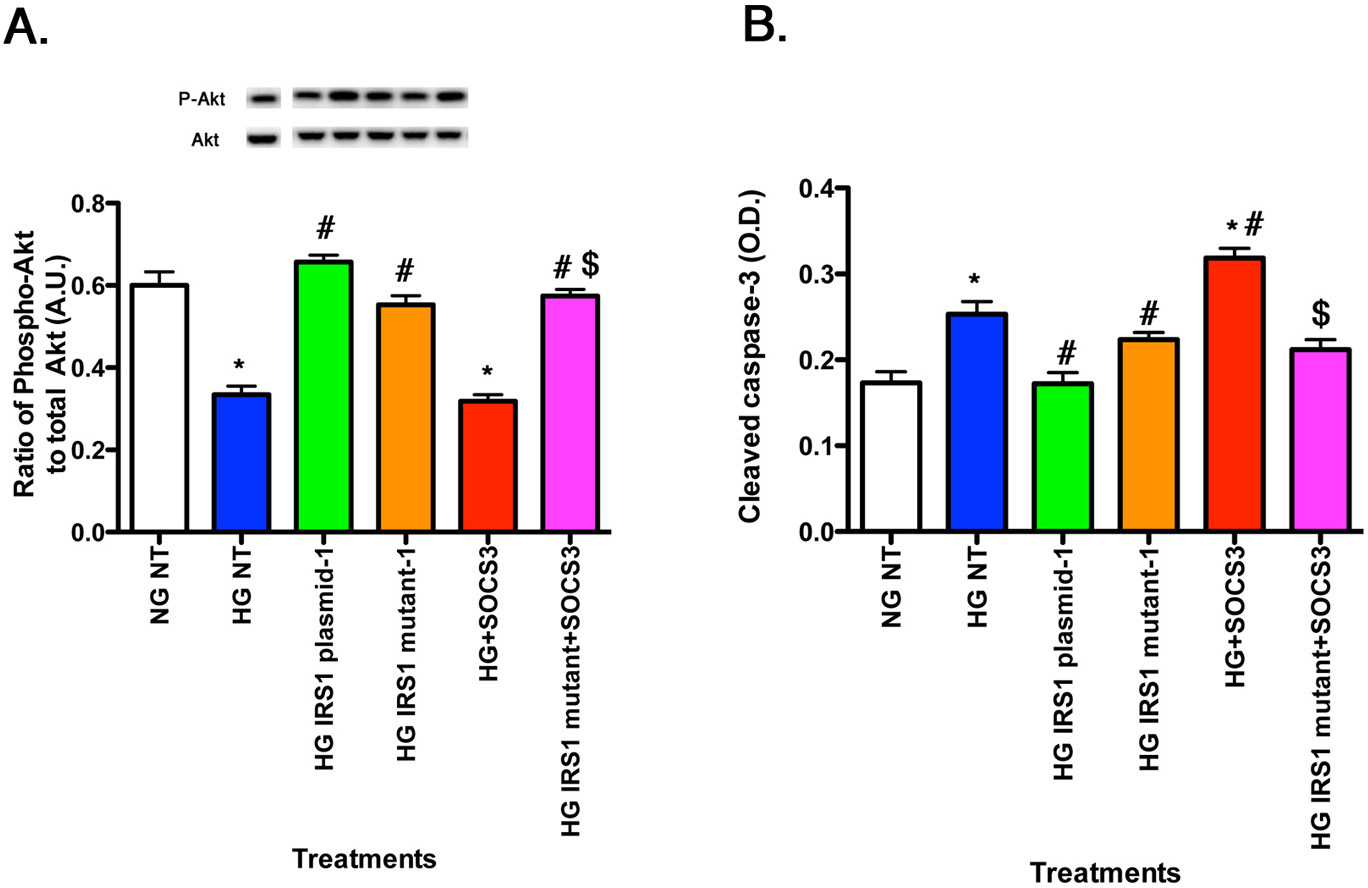
For all panels, rMC-1 cells were grown in normal glucose (NG) or high glucose (HG) and transfected with an IRS-1 plasmid or a mutated IRS-1 plasmid and treated with SOCS3 and SOCS3 with mutant plasmid (Panels **A**–**B**) with retinal cell lysates used for all experiments. Data show that SOCS3 regulates Akt (**A**) through IRS-1^Ser307^, leading to decreased cleaved caspase 3 (**B**) when IRS-1^Ser307^ is mutated. *p<0.05 versus NG non-treated; #p<0.05 versus HG non-treated; $p<0.05 versus HG SOCS3. n=5 for all groups. Data are mean±standard error of the mean (SEM).

Exogenous SOCS3 reduced Akt phosphorylation (Figure 7A), leading to increased cleaved caspase 3 levels, suggesting that SOCS3 acts to promote insulin resistance. However, when SOCS3 was added following IRS-1^Ser307Ala^ transfection, the Akt levels were significantly increased compared to SOCS3 alone (Figure 7A, magenta bar), while cleaved caspase 3 levels were reduced (Figure 7B, magenta bar). Taken together, these data suggest that TNF-α and SOCS3 required an active IRS-1^Ser307^ site to induce insulin resistance and mutation of this site eliminated the actions of both proteins to increase cleavage of caspase 3.

## Discussion

The major finding of this study is that TNF-α signaling pathways triggered by high glucose can be blocked by a single alanine substitution at a key phosphorylation site, serine 307, on IRS-1. Furthermore, the full complement of the downstream effects of TNF-α signaling pathways, including the inhibition of insulin receptor binding to IRS-1 as well as the antiapoptotic factor, phosphorylated Akt, and the stimulation of the proapoptotic factor, cleaved caspase 3, are blocked by the alanine-substituted form of IRS-1. We chose to target the serine 307 locus–based on previous studies that show 1) TNF-α preferentially phosphorylates IRS-1^Ser307^ to inhibit insulin signal transduction [5,16] and 2) a decrease in TNF-α levels (in response to treatment with salmeterol, a β2-adrenergic receptor agonist) reduces retinal rMC-1 cell apoptosis [4]. Our new findings suggest that TNF-α-stimulated phosphorylation of the IRS-1 serine 307 locus is a major driving force in the deleterious effects of high glucose on rMC-1 cells. Increased phosphorylation of IRS-1 would be expected to directly inhibit binding of the insulin receptor to IRS-1 and thus block normal insulin stimulation of its receptor, causing insulin resistance. The pathway by which TNF-α changes the balance between the levels of anti- versus proapoptotic factors is as yet, unclear, although IRS-1 phosphorylation appears to be key.

A somewhat unexpected finding from these experiments is the high degree to which SOCS3 partners with TNF-α in affecting high-glucose stress responses in rMC-1 cells. It has been previously shown that SOCS3 inhibits insulin signaling through multiple pathways, including phosphorylation of IR^Tyr960^, which inhibits insulin receptor/IRS-1 interaction, and ubiquinitization of IRS-1, which leads to IRS-1 destruction [8,9,17]. Our data demonstrate that, like TNF-α, SOCS3 increases phosphorylation of IR^Tyr960^ in rMC-1 cells grown in high glucose, and this leads to a concomitant decrease in antiapoptotic Akt phosphorylation and an increase in proapoptotic cleavage of caspase 3. In addition, we report for the first time that IRS-1^Ser307^ is required for SOCS3 actions on IR^Tyr960^, as this does not occur if IRS-1^Ser307^ is mutated. Thus, TNF-α and SOCS3 require an active serine 307 phosphorylation site on IRS-1 to bring about insulin resistance.

Another novel observation in this study is that SOCS3 can increase TNF-α levels. We find that rMC-1 cells in high glucose have increased TNF-α, which is further stimulated with the addition of exogenous SOCS3. These results are consistent with the report that silencing SOCS3 inhibits TNF-α levels in preadipocytes [7]. The reciprocal stimulation of SOCS3 and TNF-α expression suggests that positive feedback between these two factors could play an important role in mediating the development of insulin resistance and apoptotic cell death in rMC-1 cells.

In conclusion, TNF-α and SOCS3 inhibit insulin signaling and stimulate retinal rMC-1 cell apoptosis; both mechanisms act through phosphorylation of IRS-1^Ser307^. Increases in TNF-α and SOCS3 are triggered by high glucose and through reciprocal stimulation of the expression of these two factors, which in turn could be major drivers of insulin resistance and related cell death. The demonstration that a single phosphorylation site is key for these pathways suggests that drugs targeted to this site might be effective in protecting against diabetic damage to the retina.
